# Microstructure and Properties of Heat Affected Zone in High-Carbon Steel after Welding with Fast Cooling in Water

**DOI:** 10.3390/ma13225059

**Published:** 2020-11-10

**Authors:** Michail Nikolaevich Brykov, Ivan Petryshynets, Miroslav Džupon, Yuriy Anatolievich Kalinin, Vasily Georgievich Efremenko, Natalia Alekseevna Makarenko, Danil Yurievich Pimenov, František Kováč

**Affiliations:** 1 Welding Department, Zaporizhzhia Polytechnic National University, 69063 Zaporizhzhia, Ukraine; 2 Institute of Materials Research, Slovak Academy of Sciences, 04001 Kosice, Slovakia; ipetryshynets@saske.sk (I.P.); mdzupon@saske.sk (M.D.); fkovac@saske.sk (F.K.); 3 PrJSC Zaporozhtransformator, 69600 Zaporizhzhia, Ukraine; kalininurij16@gmail.com; 4 Physics Department, Pryazovskyi State Technical University, 87555 Mariupol, Ukraine; vgefremenko@gmail.com; 5 Welding Department, Donbass State Engineering Academy, 84313 Kramatorsk, Ukraine; sp@dgma.donetsk.ua; 6 Department of Automated Mechanical Engineering, South Ural State University, Lenin Prosp. 76, Chelyabinsk 454080, Russia; danil_u@rambler.ru

**Keywords:** high-carbon steel, welding, fast cooling, water, tensile tests, austenite, martensite, bainite

## Abstract

The purpose of the research was to obtain an arc welded joint of a preliminary quenched high-carbon wear resistant steel without losing the structure that is previously obtained by heat treatment. 120Mn3Si2 steel was chosen for experiments due to its good resistance to mechanical wear. The fast cooling of welding joints in water was carried out right after welding. The major conclusion is that the soft austenitic layer appears in the vicinity of the fusion line as a result of the fast cooling of the welding joint. The microstructure of the heat affected zone of quenched 120Mn3Si2 steel after welding with rapid cooling in water consists of several subzones. The first one is a purely austenitic subzone, followed by austenite + martensite microstructure, and finally, an almost fully martensitic subzone. The rest of the heat affected zone is tempered material that is heated during welding below A_1_ critical temperature. ISO 4136 tensile tests were carried out for the welded joints of 120Mn3Si2 steel and 09Mn2Si low carbon steel (ASTM A516, DIN13Mn6 equivalent) after welding with fast cooling in water. The tests showed that welded joints are stronger than the quenched 120Mn3Si2 steel itself. The results of work can be used in industries where the severe mechanical wear of machine parts is a challenge.

## 1. Introduction

Welding is one of the oldest technologies in the world. Ancient weapons made more than a thousand years ago can serve as the evidence of how old the welding technology is [1]. Nowadays, welding plays an outstanding role in industry and is used in such sectors as construction and building, transportation, energy, and others [2,3]. An important field in which welding technologies are widely used is the manufacturing and renovation of wear-resistant machine parts.

Friction and wear cause ~23% of total energy consumption in the world [4]. Significant costs are spent for remanufacturing worn parts and spare equipment. Wear is considered to be more critical than friction as it may cause catastrophic failures and breakdowns of machine operations. The mining sector is one that is influenced by wear the most compared to other industries [5,6].

Among all types of wear, abrasive wear causes the highest rate of material removal from the friction surface. The losses due to abrasive wear are estimated at 4% of the gross national product in industrially developed countries [7].

There are a number of ways to mitigate problems connected with abrasive and other types of severe wear. Arc weld surfacing [8,9,10,11] is an effective method to reduce wear losses. Wear resistant materials may be also used to produce the entire working unit [12,13,14,15]. Finally, it is possible to protect certain critical places of a machine part with elements made of wear resistant composition [16].

Iron-based alloys (steels and cast irons) are widely used as wear resistant materials. As a rule, they are subjected to heat treatment in order to increase wear resistance. Usually, the quenching process is applied to form the structure appropriate for specific working conditions [17]. If after quenching the material is intended for working as a wear resistant element, then welding may be used to attach such an element to the base machine part. 

The problem is that high heat input during welding will disrupt the wear resistant structure that is obtained by quenching. Moreover, wear resistant steels usually possess high a carbon equivalent [18,19] that significantly lowers weldability [20,21]. Therefore, preliminary and accompanying heating should be used in the course of welding, which negatively affects the initial structure.

Presently, high-carbon steels are mainly welded by friction welding [22,23], friction stir welding [19,24,25,26], or laser welding [27]. Arc welding is not applied for this purpose because of the cracking of the welded joints. Arc welding is favorable due to its simplicity in comparison with the above-mentioned welding techniques. Therefore, an appropriate arc welding technology should be developed to be used to safely join the quenched high-carbon steels. Also, this technology should provide the retention of initial structure obtained by the heat treatment.

Recently, a new high-carbon low-alloy wear resistant steel 120Mn3Si2 was proposed and investigated in abrasive wear conditions [28,29]. It was shown that the high wear resistance of this steel can be obtained after quenching from temperatures in the range of 800–1000 °C. 

The following considerations were taken into account for welding the quenched 120Mn3Si2 steel. The structure of material in a heat affected zone (HAZ) depends on the distance from the fusion line and on cooling rate. If fast cooling is used instead of slow cooling, then each HAZ layer heated above A_1_ critical temperature will be quenched.

**Figure 1:**
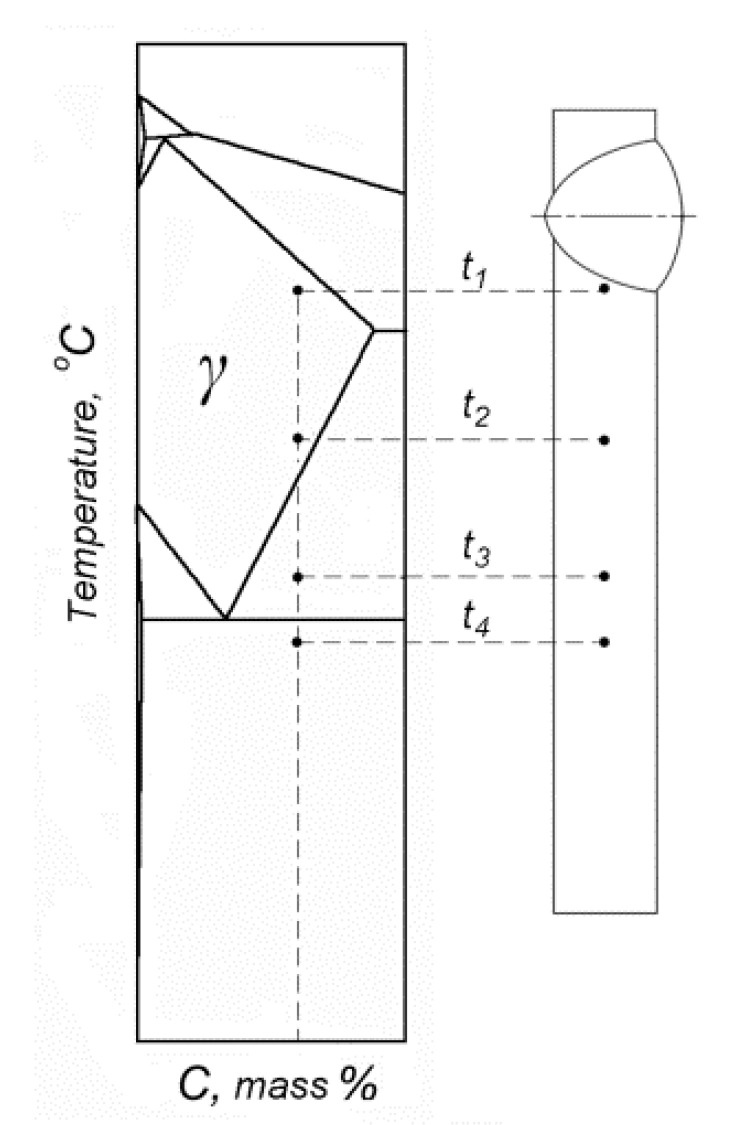
Schematic representation of different heating points in a heat affected zone (HAZ) during welding of 120Mn3Si2 steel in accordance with phase diagram adapted from [30].

A phase diagram for corresponding welded material may be used to predict the structure of HAZ. According to the phase diagram for 2.5% Mn alloy by Houdremont [30], steel with 2.5% Mn and 1.2% C (designated as 120Mn3Si2) can be quenched from single-phase γ-region in the near vicinity of the fusion line. This situation corresponds to temperatures from t_1_ to t_2_ on Figure 1. It is obvious that fast cooling from t_3_ will result in a predominantly martensitic structure formation; fast cooling from t_4_ will not result in phase transformation at all. Therefore, a sharp drop in hardness is expected between HAZ layers corresponding to t_3_ and t_4_. The question remains, which structure would appear in HAZ between points corresponding to temperatures from t_1_ to t_2_? 

According to the theory of heat treatment, the structure after quenching depends on the martensite start temperature (Ms). Ms, in turn, depends on the chemical composition of austenite just before cooling. When 120Mn3Si2 steel is heated to a single-phase region, all carbon and alloy elements should dissolve in austenite. Therefore, the chemical composition of austenite will be the same as that for steel. This causes Ms to be at 30 °C and, therefore, leads the formation of an almost fully austenitic structure after quenching from single-phase region [28,29]. As a result, a hard martensitic layer in HAZ (quenching from t_3_) should be surrounded by two much softer layers: an austenitic layer from one side (quenching from t_1_ to t_2_) as well as a layer of highly tempered material (heating to t_4_).

Thus, it is expected that a fully austenitic layer will appear in the near vicinity of the fusion line after welding with rapid cooling [31]. The width of this and all subsequent layers in HAZ depend on cooling rate. The higher cooling rate, the less width of HAZ is expected.

Cooling in water right after welding is the simplest way to ensure a fast cooling rate. There are some other methods that can be used for even faster cooling [27,32], but they are much more complicated. The approach of applying water cooling in relation to arc welded high-carbon steel has not been studied yet. Therefore, the aim of the present work was to evaluate the structural features of pre-quenched 120Mn3Si2 steel subjected to arc welding with further immediate water cooling.

## 2. Materials and Methods

120Mn3Si2 steel was used for the welding experiments. The chemical composition was as follows (wt.%): 1.21 C; 2.56 Mn; 1.59 Si; 0.25 Cr; 0.10 Ni; 0.01 P; 0.01 S; balance is Fe. The steel was melted in a vacuum furnace. The castings were then forged and rolled down to 300 × 60 × 5 mm strips. The strips were decarburized to a depth of approximately 1 mm. The carbon concentration on the very surface of strips was about 0.8 wt.% C. The UTS of as-manufactured strips was 820 ± 20 MPa. These strips were used to cut off samples for heat treatment and welding experiments. The reason behind the necessity of surface decarburization in 120Mn3Si2 steel is discussed further.

Before quenching, the samples were heated in a LAC PKE Hardening chamber (LAC, s.r.o., Židlochovice, Czech Republic) furnace with a Ht40P controller. Water was the quenching medium. The microstructures of quenched material and HAZ of welding joints were observed in JEOL JSM-7000F scanning electron microscope (Jeol Ltd., Tokyo, Japan) and Olympus GX71 optic microscope (OLYMPUS Europa Holding GmbH, Hamburg, Germany). Vickers hardness and microhardness were measured by a Wilson^®^ Hardness tester (Wolpert Wilson Instruments, Division of INSTRON DEUTSCHLAND GmbH, Aachen, Germany). 

ISO 4136 tensile tests were carried out for welded joints of 120Mn3Si2 steel and 09Mn2Si low carbon steel (ASTM A516, DIN13Mn6 equivalent) in the final stage of the investigation. Tensile tests were performed by an INSTRON 250 machine (Instron Worldwide Headquarters, Norwood, MA, USA).

## 3. Results and Discussion

**Figure 2:**
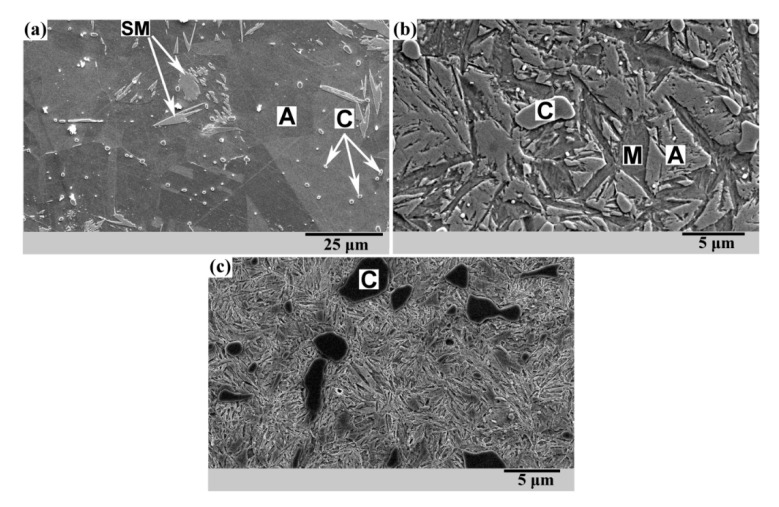
Microstructure of 120Mn3Si2 steel after quenching from different temperatures. Temperature of heating: (**a**) 1000 °C, ×1000; (**b**) 900 °C, ×4000; (**c**) 800 °C, ×3700.

Samples were quenched from three different temperatures: 1000 °C, 900 °C, and 800 °C. As a result, structures with a different amount of retained austenite were obtained after quenching. Figure 2 shows SEM micrographs of steel 120Mn3Si2 after quenching from 1000 °C, 900 °C, and 800 °C.

After quenching from 1000 °C, the structure was predominantly austenitic (A) containing a small amount of undissolved carbides (C) (Figure 2a). Due to the instability of the retained austenite, separate fields of surface martensite (SM) were observed. Surface martensite appears as thin plates during grinding/polishing in the process of sample preparation. The hardness of the material after quenching is 220–240 HV0.5.

Quenching from 900 °C provides a microstructure that differs significantly (Figure 2b). Undissolved carbides are much coarser. A significant amount of martensite is also present. This is due to Ms that is higher than that for quenching from 1000 °C. The rise of the Ms is caused by the lower carbon concentration in austenite at 900 °C than that at 1000 °C. The hardness of the material after quenching is 480–500 HV0.5.

A further decrease of quenching temperature down to 800 °C leads to a microstructure of hardened hypereutectoid steel (Figure 2c): a predominantly martensitic matrix with coarse undissolved carbides. The hardness of the material after quenching is 790–800 HV0.5.

The welding of 120Mn3Si2 steel quenched from 800 °C was the first step of the experiments. Because of the highest hardness of the base material (i.e., 790–800 HV0.5) the most explicit boundary between HAZ and thermally unaffected structure was expected. Thus, samples of 120Mn3Si2 steel quenched from 800 °C were welded with conventional 09Mn2Si steel (ASTM A516 grade steel intended for welded constructions). In this experiment 09Mn2Si steel represents a wide class of low-carbon constructional steels with good weldability but low wear resistance.

The thickness of the 09Mn2Si plate was 7 mm. The plates of welded steels were clamped against each other and submerged into water. The welding edges were 5–10 mm higher than the water level. MMA welding of the plates (lap joint) was performed with a AS Pik-98 Süper electrode (1% of C; 99% of Ni; Askaynak, Turkey) 3 mm in diameter. An alternating current of 190 A, 25 V was used. The welding speed was approximately 1.5 mm/s.

**Figure 3:**
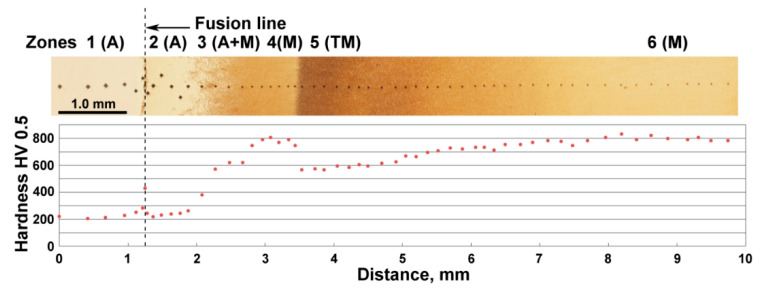
Microstructure and hardness of HAZ of 120Mn3Si2 steel after welding with fast cooling in water. A—austenite; M—martensite; TM—tempered martensite.

Figure 3 shows the change of macrostructure and hardness of welded 120Mn3Si2 steel from weld metal through HAZ to thermally unaffected material. Several zones may be distinguished here. Zone 1 (weld metal) should have austenitic structure (A) according to the chemical composition of the electrode. The hardness of this zone is about 200–220 HV0.5, which corresponds to that of austenite. Zone 2–6 represent welded 120Mn3Si2 steel. Zone 2 has austenitic (A) structure with the same hardness as well. This zone corresponds to quenching 120Mn3Si2 from temperatures between t_1_ to t_2_ (see Figure 1). Zone 3 comprises mixed austenite + martensite (A + M) structure and corresponds to quenching from temperatures between t_2_ and t_3_. The hardness in zone 3 gradually increases with the distance from the fusion line. Hardness reaches its maximum about 800 HV0.5 at approximately 1.6 mm from the fusion line. Zone 4 with preliminary martensitic (M) structure begins here and is ~0.5 mm thick. This zone corresponds to quenching 120Mn3Si2 steel from t_3_. At the end of zone 4 a sharp drop in hardness can be seen. This is due to the transfer from zone 4 (quenching during welding) to zone 5 where the structure consists of tempered martensite (TM). Zone 5 is about 3.5 mm wide and gradually transforms into zone 6 with the structure of martensite (M) which is not affected by the welding heat.

**Figure 4:**
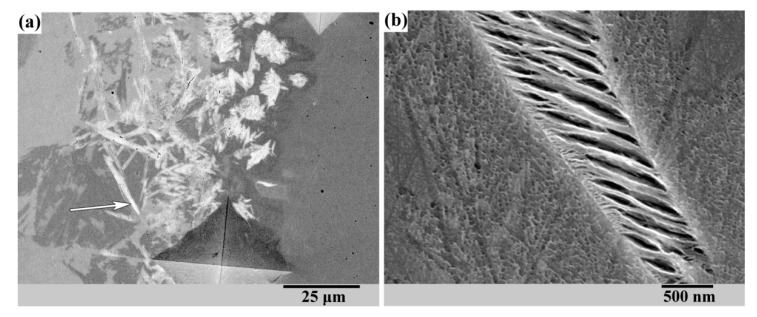
SEM micrographs of martensite inside fusion line in different magnification: (**a**) ×1000; (**b**) ×33,000.

The transformation of the structure and hardness in zones 1 to 6 is in accordance with the considerations outlined above (see Figure 1). The only difference is the sharp peak of hardness just at the fusion line. Figure 4a shows the microstructure that is observed along the width of the fusion line.

The microstructure suggests that a certain amount of martensite appears inside the fusion line. Figure 4b represents a closer view of one of the martensite plates (see arrow on Figure 4a). According to the microstructure shown in Figure 4b, the martensite is tempered obviously due to the welding heat. This may be the reason why no cracks are observed in and near the fusion line despite a decent amount of martensite and quite high hardness. 

Martensite in the fusion line after welding with rapid cooling of 120Mn3Si2 steel was also observed in [31]. The supposed reason was the local rising of Ms presumably due to the decarburizing (“dilution”) of base metal under melting of electrode material. The assumption was that increasing the content of austenite-forming elements in the electrode would eliminate the dilution of the base metal and thus the formation of martensite inside the fusion line. This is why a nickel electrode was used for the experiment presented here. However, martensite still appears in the fusion line despite the high nickel content in electrode material. This leads to the conclusion that martensite in the fusion line cannot be eliminated even if the electrode material contains 100% austenite-forming elements.

**Figure 5:**
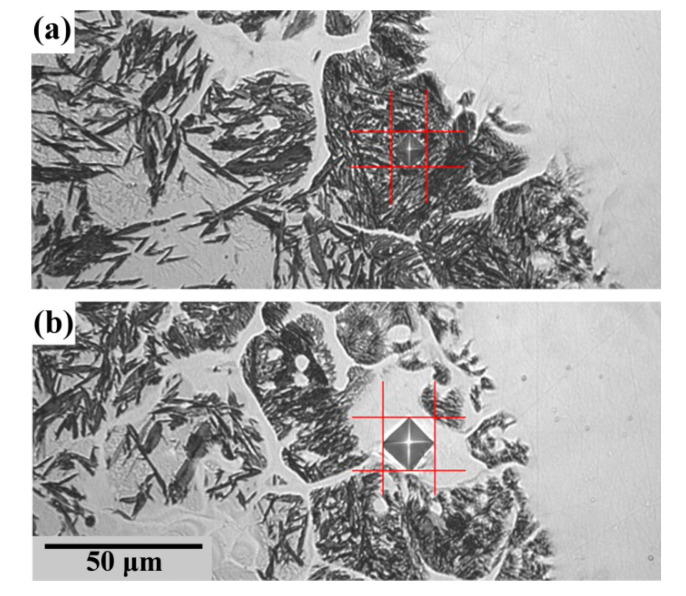
Indents after microhardness measurement of unmelted grain (**a**) and remelted metal (**b**). Values of microhardness: (**a**) 740 HV0.05; (**b**) 329 HV0.05.

Another observation is that martensite inside the fusion line appeares as isolated “grains” surrounded by austenitic bands (Figure 5). This corresponds with the fact that fusion line is consisted of partially melted grains of base material. These grains are surrounded by remelted metal which is a mixture of base and electrode materials. 

Figure 5 shows indents after microhardness measurement of unmelted grain (Figure 5a) and surrounding remelted metal (Figure 5b). Martensite (see indent on Figure 5a) seems to appear inside the unmelted parts of the grains. These unmelted parts cannot be diluted by electrode metal. Therefore, the reason for martensite’s appearance inside unmelted grains in the fusion line cannot be connected with the composition of the electrode.

Whatever the reason for martensite appearance in the fusion line was, it did not cause cracks in a welding joint. This is possibly due to the tempering of martensite by welding heat and soft austenite interlayers of melted metal surrounding the unmelted grains.

**Figure 6:**
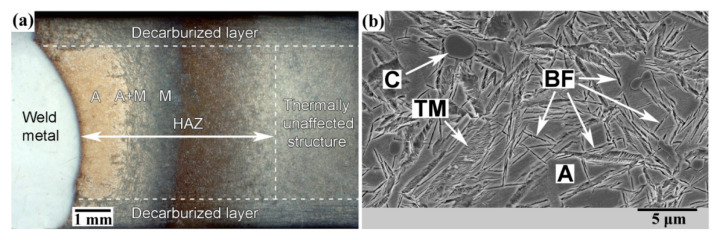
(**a**) Macrostrusture of HAZ of 120Mn3Si2 steel after welding with rapid cooling in water; (**b**) One of the possible thermally unaffected structures—quenching from 900 °C and isothermal treatment at 250 °C during 2 h, ×4000. A—austenite; C—carbides; TM—tempered martensite; BF—bainitic ferrite.

Figure 6a shows the typical macrostructure of a quenched 120Mn3Si2 steel sample welded with rapid cooling in water. A decarburized layer about 1 mm in depth is present on both sides of the welded sample. The microstructure here would be preliminary martensitic either in HAZ or in a thermally unaffected zone.

Three zones are presented in HAZ which are analogous to zones 2 to 4 from Figure 2. These zones are located in the core of 120Mn3Si2 sample between the decarburized layers and appear after welding independent of the previous thermal treatment of 120Mn3Si2 steel. The zones are austenite (A), austenite + martensite (A + M), and martensite (M).

The thermally unaffected structure depends on the preliminary thermal treatment of 120Mn3Si2 steel. Three different possible microstructures are shown in Figure 2. Quenching from 800 °C (see Figure 2c) results in a predominantly martensitic microstructure of high hardness and high brittleness, and therefore it is unlikely to be used in practice. Quenching from 1000 °C and 900 °C (see Figure 2a,b) results in a less brittle microstructures which possess even higher abrasive wear resistance than the predominantly martensitic one [29]. Quenching from 900 °C is more favorable than quenching from 1000 °C because it provides slightly higher abrasive wear resistance [29] and accelerates bainite transformation at 250 °C [28]. The acceleration of bainite transformation is caused by pre-existing martensite [33,34,35] which is present in the microstructure after quenching from 900 °C (see Figure 2b).

Figure 6b shows the typical microstructure of 120Mn3Si2 steel after quenching from 900 °C and isothermal treatment at 250 °C during 2 h. The microstructure contains austenite A, carbides C, tempered martensite TM, and thin plates of bainitic ferrite BF. This microstructure is significantly more ductile than that without isothermal holding (see Figure 2b) [28] and has abrasive wear resistance as high as untempered martensite after quenching from 800 °C [29]. Thus, a combination of quite high abrasive wear resistance (like untempered martensite) and significantly higher ductility of lower bainite [36,37,38] makes this microstructure the most favorable for application in abrasive wear conditions.

The question remains whether the decarburized layer should be considered as a defect for wear resistant plates made of 120Mn3Si2 steel. 

There are plenty of possible wear modes that may take place in the real application of a wear resistant part. It may be even the case that different wear modes, and hence different wear values, appear on different areas of a given friction surface [39].

Unstable austenite has high wear resistance in abrasive wear conditions [28,29,40,41,42,43,44]. This is due to mechanically induced martensite that appears from austenite on the surface during abrasive wear. However, this effect is possible only if contact stresses are sufficient for the plastic deformation of metal; this is the so-called low-cycle fatigue mechanical wear. If contact stresses are insufficient for plastic deformation, then austenite possesses lower wear resistance than as-quenched martensite; this is high-cycle fatigue mechanical wear. Retained austenite is beneficial as wear resistant microstructure if contact stresses exceed a certain threshold [45].

Thus, there are two possible modes of mechanical wear: low-cycle fatigue and high-cycle fatigue. If low-cycle fatigue is the case, then unstable austenite is more wear resistant than martensite. If the machine part works in high-cycle fatigue conditions, then the martensite possesses higher wear resistance than unstable austenite. In any case, wear resistant materials should possess high fatigue resistance [11].

In practice, a friction surface can work under any of two possible modes of mechanical wear or even under both of them. Therefore, it would be rational to provide both predominantly martensitic and predominantly austenitic structures inside the wear resistant element. This goal is precisely achieved in a 120Mn3Si2 steel plate with decarburized layers (see Figure 6a). The following is the rationale behind this statement.

Suppose that the wear mode is high-cycle fatigue (relatively low contact stresses). The wear rate (or wear intensity) in high-cycle fatigue mode is several orders of magnitude lower than that in a low-cycle fatigue mode. Therefore, 1 mm of a predominantly martensitic layer would work for a significant time until it becomes worn. After wearing of this layer, the predominantly austenitic core will start working. Although not so high in wear resistance as martensite [45], austenite still possesses satisfactory resistance during high-cycle fatigue wear. Therefore, the austenitic core will provide satisfactory working time after wearing of the top martensitic layer. Finally, after wearing of the austenitic core, the martensitic layer located on the opposite side of plate will start working. Overall, if the wear mode is high-cycle fatigue then two martensitic layers provide excellent wear resistance, and the austenitic core possesses satisfactory wear resistance. Therefore, a significant lifetime for machine part would be achieved. 

The roles of layers would be changed in the case of low-cycle fatigue wear. Instable austenite is able to transform into mechanically induced martensite during low-cycle fatigue wear. Thus, the wear resistance of the austenitic core will be greater than that for martensitic layers [28,29,40,41,42,43,44]. Nevertheless, the wear resistance of martensitic layers would be satisfactory as well. As a result, if the wear mode is low-cycle fatigue, then an austenitic core provides excellent wear resistance, and two martensitic layers have satisfactory wear resistance. Thus, decarburizing of 120Mn3Si2 steel is a useful phenomenon. Quenching such material from 900–1000 °C provides a universal wear resistant plate for mechanical wear conditions.

**Figure 7:**
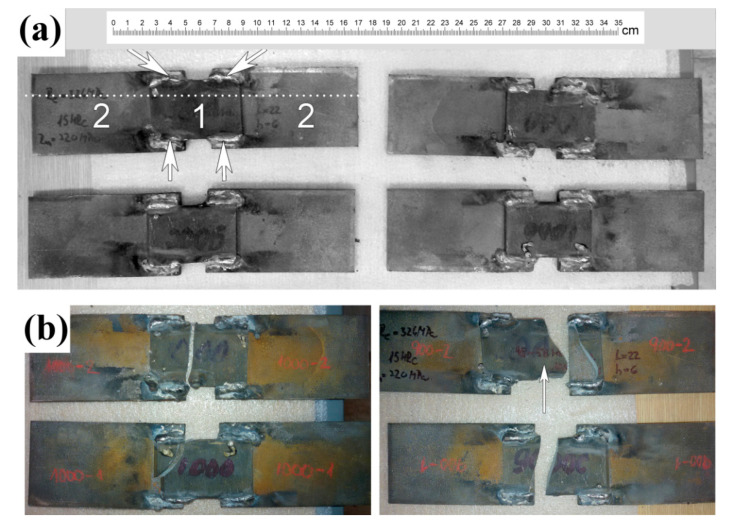
Non-standard welded samples from 120Mn3Si2 steel (1) and 09Mn3Si2 steel (2) before (**a**) and after (**b**) testing.

The next step of investigation was assessing the quality of the welding joints of quenched 120Mn3Si2 steel after welding with rapid cooling in water. Tensile tests of non-standard welded samples were performed on the first stage. The welded samples are shown in Figure 7a. Each sample consisted of two plates of 09Mn2Si steel (2) welded by four lap joints with one quenched plate of 120Mn3Si2 steel (1) (see Figure 7a). The dotted line shows the water level during welding. This form of welded samples was chosen because we needed tangential stresses in welding joints. Usually, welding joints possess lower strength when facing a tangential load compared to normal one. As such, we intended to model the worst loading case. Also, during wear the machine parts work mainly under tangential forces that cause tangential stresses. The length of plates overlapping was about 20 mm, so that was the length of the welds. The goal was to figure out if the rupture would go through weld metal or through HAZ. In addition, a rough estimation of weld strength to tangential load was expected to be obtained. 

There were four samples in total. Two plates of 120Mn3Si2 steel were quenched from 1000 °C, and other two were quenched from 900 °C. 

The samples appearance after tensile test is shown in Figure 7b. It should be noticed that all four samples were broken through 120Mn3Si2 quenched plate, not through welds.

**Figure 8:**
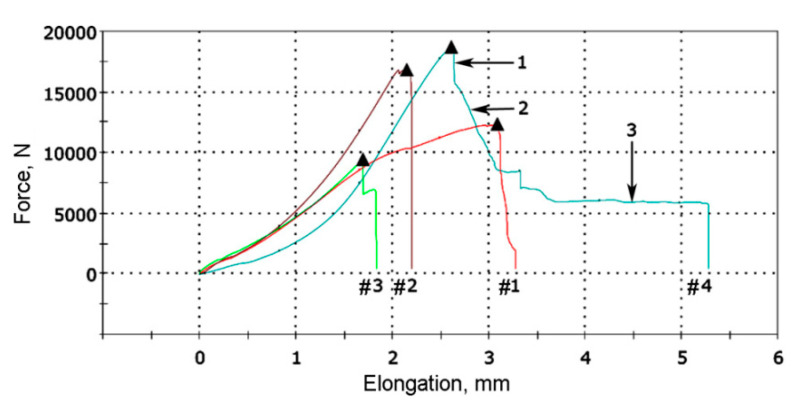
Loading diagram for non-standard welded samples.

The loading diagrams for all four samples are shown in Figure 8. The plates of 120Mn3Si2 steel for samples #1 and #2 were quenched from 1000 °C. The plates for samples #3 and #4 were quenched from 900 °C. The minimal and maximal breaking loads recorded were 9488 N and 18,690 N for samples #3 and #4, respectively. In this way, no significant difference in breaking load was noticed between plates quenched from different temperatures. Another output was that the breaking load appeared an order of magnitude lower than expected. The cross-section of quenched 120Mn3Si2 plates was approximately 200 mm^2^. Therefore, nominal stresses in the base material at the moment of breakage were 47 MPa (9488 N load) and 93 MPa (18,690 N load).

This fact may be explained by the gradient structure of cross-section of decarburized 120Mn3Si2 plate after quenching. During loading, the stress is concentrated in surface martensitic layers. After initial brittle damage of this layer a crack appears, therefore, stresses become concentrated further at the crack tip. Plastic deformation of retained austenite at the crack tip leads to γ → α transformation, and freshly formed brittle martensite cracks as well. This chain process (i.e., crack → deformation → transformation → crack) proceeds at a relatively low load until the entire plate is broken.

**Figure 9:**
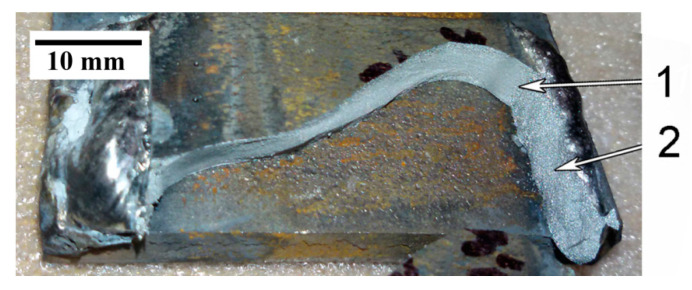
Broken sample #4: (1) core of broken 120Mn3Si2 plate; (2) weld that carried the load in the last stage of failure.

The loading diagram for sample #4 (see Figure 7, arrow; Figure 8) confirms the assumptions given above. Arrow 1 on Figure 8 indicates a sharp drop in load because of initial crack. The crack stops at a certain depth and some plastic deformation of the core takes place (arrow 2). The horizontal site (arrow 3) corresponds to the interesting moment. The crack reaches the weld (arrow 1 on Figure 9) and the austenitic layer bears the load during a certain time with concomitant plastic deformation.

Since no one sample was broken in the weld metal (see Figure 7), ISO 4136 standard tests were performed in order to assess tensile strength of welding joints. The standard welded samples were 5 mm thick. One part of the sample was 120Mn3Si2 steel without heat treatment, the other one was 09Mn2Si steel. Eight samples were prepared. The samples were plunged into water by the 120Mn3Si2 side during welding.

**Figure 10:**
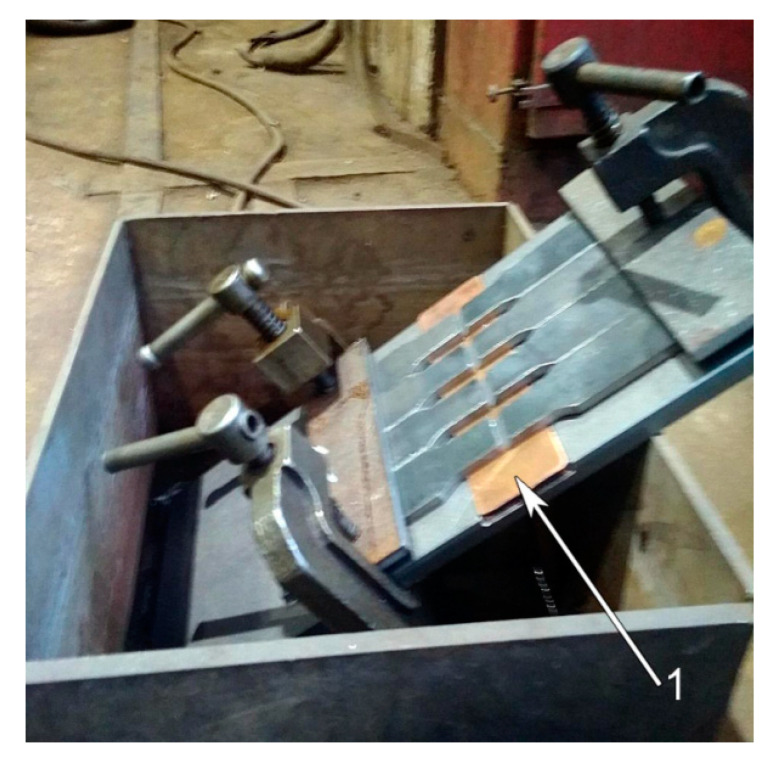
Standard welded samples clamped before welding.

Figure 10 shows four clamped samples which are located in the reservoir ready for welding. A copper plate (arrow 1) was initially intended to facilitate cooling. It was found that that the water reached the welding arc via gap between the sample and copper plate because of surface tension. Therefore, the copper plate was not used in the further experiments.

Table 1 shows the results of the testing of welded samples. According to these results, the UTS of welded samples is 209 ± 27 MPa. All samples were broken via HAZ on 120Mn3Si 2 sides via the martensitic layer. The relatively low UTS may be explained by high carbon untempered martensite which forms this layer. Despite its low value, the UTS is higher than that for quenched 120Mn3Si2 plate (about 90–100 MPa, typical for ultrahigh carbon martensite [46]). Therefore, welding joints may be used even without subsequent thermal treatment, since wear resistant plates are usually not subjected to normal breaking loads. If in some cases UTS appears insufficient, the welding joints may be subjected to usual temper which can increase strength. Future research should be conducted to investigate the influence of post-welding heat treatment on microstructure and mechanical properties of 120Mn3Si2 steel welding joints.

## 4. Conclusions

In this study 120Mn3Si2 steel was been welded with rapid cooling in water. Welding joints of sufficient quality were obtained. The following conclusions can be drawn:

1. Quenched 120Mn3Si2 steel may be successfully welded with rapid cooling in water. An austenitic layer is formed in the vicinity of the fusion line followed by mixed austenite– martensite structure, and finally, the layer of hard untempered martensite.
2. Despite the presence of a hard martensitic layer, there were no cracks observed in HAZ. This is possibly due to a ductile austenitic layer separating themartensite from the fusion line.
3. A noticeable amount of martensite appears inside the fusion line despite the highly austenitic weld metal. This martensite is located in the unmelted grains of 120Mn3Si2 steel which are surrounded by remelted austenitic metal. SEM observation indicates typical structure of tempered martensite. Thus, martensite in the fusion line does not cause damage to the weld due to its temper and ductile austenitic metal which surrounds partially remelted grains.
4. The UTS of welding joints 209 ± 27 MPa were for 120Mn3Si2 steel samples as a result of ISO 4136 tests. Further research is needed to find ways to increase the UTS of the welding joints of high-carbon steels by appropriate thermal or other treatments.

## Figures and Tables

**Table 1:** Results of ISO 4136 tests of 120Mn3Si2 and 09Mn2Si steels welded with rapid cooling in water.

Sample #	Maximal Load, N	UTS, N/mm^2^	Location of Damage
1	32,634	236	HAZ of steel 120Mn3Si2
2	25,186	195	
3	31,948	231	
4	20,972	152	
5	31,360	232	
6	22,638	174	
7	37,730	290	
8	21,560	161	
